# A Manual of Medical Jurisprudence and Toxicology

**Published:** 1897-10

**Authors:** 


A Manual of Medical Jurisprudence and Toxicology—By Henry C. Chapman, M. D., Professor of Medical Jurisprudence in the Jefferson Medical College, etc., etc. Second edition, revised. With 55 illustrations and 3 plates in colors. W. B. Saunders, Publisher, Philadelphia. Pp. 254. 1896.

This is one of the best manuals on the subject of medical jurisprudence and toxicology. It has passed through its first ddition, this one being the second; and students have found it very satisfactory as a text book. It is well arranged and covers in a concise way all the practical subjects embraced in a work of this kind. The author is to be congratulated on getting so much useful information into so small a space. H.

